# Sexual & reproductive health information on minor consent forms for pubertal suppression and gender affirming hormones

**DOI:** 10.3389/frph.2023.1071212

**Published:** 2023-04-19

**Authors:** Julia Taylor, Meesha Vullikanti, Samhita L. Nelamangala, Katherine E. Boguszewski, Mary Faith Marshall

**Affiliations:** ^1^ Center for Health Humanities & Ethics, Univerisity of Virginia, Charlottesville, VA, United States; ^2^ School of Medicine, Department of Pediatrics, University of Virginia, Charlottesville, VA, United States; ^3^ Department of Obstetrics and Gynecology, Vanderbilt University Medical Center, Nashville, TN, United States

**Keywords:** transgender, sexual health, informed consent, pubertal suppression, gender affirming hormone

## Abstract

**Introduction:**

Transgender and Nonbinary (TNB) youth need specialized sexual and reproductive health (SRH) information and counseling. One avenue for providing this information is the use of informed consent documents before initiating pubertal suppression (PS) and/or gender-affirming hormones (GAHs). This study aims to compare the type and amount of SRH information included on informed consent documents used across clinical sites providing PS and GAH to youth.

**Methods:**

As part of a larger, IRB-approved survey on informed consent, providers of gender-related care to youth uploaded informed consent forms used in clinical practice. Publicly available forms were also included in analysis. Content analysis of these forms was undertaken using published clinical guidelines to inform coding and reflect the SRH implications of starting PS and GAH.

**Results:**

21 unique consent documents were included in the content analysis (PS = 7, Masculinizing = 7, Feminizing = 7). SRH information on consent documents fell into 4 broad categories: (1) changes in sexual organs and functioning; (2) pregnancy and fertility information; (3) cancer risk; and (4) sexually transmitted infections. Forms varied considerably in the level of detail included about these SRH topics and most forms included implicit or explicit acknowledgement of the uncertainty that exists around certain SRH outcomes for TNB youth.

**Conclusions:**

There was substantial variability in both SRH content and context across consent forms. The role of consent forms in fostering TNB youth's understanding of complex SHR information when initiating PS and GAHs needs further clarification and development. Future research should focus on ways to ensure provision of adequate SRH information for TNB youth.

## 1. Introduction

Transgender and Non-binary (TNB) youth need access to specialized sexual and reproductive health (SRH) information and counseling. Studies describing the sexual and reproductive health needs of TNB youth report variable rates of contraceptive use (1–3), pregnancy involvement (4), sexually transmitted infections (3, 5, 6), and fertility desire (7–11), leaving TNB youth at risk for SRH disparities. Clinical guidelines for the care of TNB youth (12, 13) provide detailed information about the possible effects of medical interventions (pubertal suppression (PS) and gender affirming hormones (GAHs)) on current and future SRH, including changes in sexual function, menstrual patterns, fertility, and cancer risk. SRH information for TNB youth is therefore expected to include both the sexual health concepts important to the care of all adolescents (e.g., contraception, sexually transmitted disease prevention) (14) as well as detailed information about the possible adverse effects of gender affirming medications on sexual and reproductive functioning (12, 13, 15).

Clinical guidance documents (12, 13) prioritize provision of adequate information (including SRH information) to support informed decision making before TNB youth begin gender affirming medical treatments. There is currently no standardized process for delivering SRH and other information to TNB youth seeking PB and/or GAHs, however formal informed consent forms are one method used in clinical settings for both medico-legal purposes and to provide youth and families with important information before initiating PS and/or GAHs (16). While multiple methods (e.g., conversations with providers and parents, online resources, flyers, brochures, or audio-visual decision aids) likely contribute the SRH knowledge of TNB youth (17, 18), exploration of the type and amount of SRH information provided on informed consent documents provides insights into the potential benefits and limitations of using this method to convey SRH information to TNB youth seeking medical interventions. This is the first study to compare the type and amount of SRH information included on informed consent documents from a convenience sample of clinical sites providing gender-related health care to TNB youth.

## 2. Methods

### 2.1. Sample

This research was approved by the University of Virginia Institutional Review Board for Social & Behavioral Sciences. As part of a larger study, providers of gender-related care to adolescents were recruited for an online Qualtrics (19) survey *via* professional listservs and contact information obtained from public websites identifying providers of gender-related care to youth. The survey focused on informed consent practices and beliefs and included a request for respondents to upload de-identified consent forms for PS and GAHs used in the care of minors. Additionally, publicly available forms were found *via* online search by using internet search engines and keywords combinations such as “consent” and “gender-affirming hormones”, “estrogen”, “testosterone”, “pubertal suppression, or “GnRH agonist”. Duplicate informed consent documents were excluded and all consent forms were anonymized prior to analysis.

### 2.2. Data analysis and coding

We used a flexible deductive approach to content analysis (20) to analyze the available consent forms. Content analysis allows for the objective and systematic analyses of text with special attention to what is being communicating through both context and content by incorporating both quantitative (counting, frequency) and qualitative approaches to the data.

Guidance documents from the Endocrine Society (12) and the World Professional Association for Transgender Health (WPATH) (13) were used to develop deductive codes pertaining to the explicit sexual and reproductive effects of PS and GAHs identified in clinical guidelines. We chose to use a predefined set of codes and a flexible approach to coding to allow researchers to note additional content items, language, and context relevant to communicating SRH information.

Researchers (JT, MV) read and coded all consent forms individually and met regularly to discuss the data. Coding was initially completed by hand and updated using qualitative analysis software, NViVO (21) to further combine, analyze, and manage data. Content analysis was conducted on all consent forms and included comparisons of density and frequency of codes within and across groups (Pubertal Suppression (PS), Masculinizing Hormones (MH), and Feminizing Hormones (FH)) to identify content common to all consent forms as well as codes unique to a specific type of consent document (e.g., SRH information specific to only feminizing, masculinizing, or pubertal suppressing agents).

During the iterative process, the code book was further refined and additional codes were added as result of preliminary and ongoing analyses (Table 1) identifying data that could not be coded using predefined codes or required further coding into sub-codes. All consent forms were coded for overall detail as well as level of detail in their description of risks and desired effects using a combination of coding density and descriptive details. Codes for overall tone included *formal* or *relational.* This binary coding approach to tone was developed after coding revealed differences between forms that emphasized the patient or parent's acceptance of risk and responsibility and those forms that encouraged questions and discussion. Coding discrepancies were identified and resolved. In all cases, researchers reached consensus on the ultimate code used.

**Table 1:** Selected code book examples.

Code	Description	Examples
Tone—Formal	Language that describes acceptance of risk or responsibility	“I have talked to my doctor or health care team about other treatment choices and their risks and/or benefits.”
Tone—Relational	Language with educational intent and explicitly welcoming questions and further discussion	“We encourage you to take all the time you need to ask questions, read, research, and think about how hormone therapy could affect you and your life.”
Fertility preservation (FP)—Encouraging	Language that encourages FP	“Banking is encouraged for all males post puberty prior to the initiation of estrogen therapy.”
FP—Discouraging	Language that discourages FP	“The procedure is intensive, invasive, costly, and must be done through a fertility specialist. Patients who want to explore banking ova are urged to speak to a reproductive specialist.”
FP—Neutral	Language that is neither encouraging nor discouraging.	“There are a variety of options for sperm banking. Check out these two websites for more information”
Uncertainty	Language highlighting lack of/inadequate research, lack of FDA approval, variability of dosing, and variable outcomes (in terms of endpoint and timing)	“may” “How long and whether this becomes permanent is difficult to predict.” “Using these medicines to block puberty is an “off-label” use. I know this means it is not approved by the Food and Drug Administration for this specific use.”

## 3. Results

21 unique consent documents were included in the content analysis (PS = 7, MH = 7, FH = 7). documents varied in length (range = 2–10 pages, mean = 4.7, SD = 2.3), readability (Flesch-Kincaid Grade Level (6.9–12.1, mean = 10.3 SD = 1.38), and structure (use of bullet points, paragraphs, sections/headers, signature lines, and initialing). Importantly, the forms varied both within and across categories in their approach to uncertainty, level of detail, and general tone. However, four broad categories of SRH codes were identified and consistent across most consent forms: (1) changes in sexual organs and functioning; (2) pregnancy and fertility information; (3) cancer risk; and (4) sexually transmitted infections (STIs).

### 3.1. Uncertainty, detail & tone

All forms included an acknowledgement of uncertainty about the SRH effects of PS, MHs, and FHs. Uncertainty was either implied using words like “may” or “usually” or made more explicit with statements about the lack of data regarding duration or dosage required for desired results as well as the possibility of unknown side effects. Forms that were less explicit in their descriptions of risk, particularly risk of cancer and infertility, relied more heavily on language that emphasized the uncertainty of these treatments: “The feminizing effects of estrogen can take several months or longer to become noticeable, and that the rate and degree of change can't be predicted” (FH1). All PS consent forms discussed the uncertainty of desired effects and the unclear impact on future GAT secondary to limited outcome data in TNB youth: “I know that it can take several months for the medication to be effective. I know that no one can predict how quickly or slowly my child”s body will respond” (PS5).

The level of detail within forms varied greatly. Forms were categorized as having either a low or high level of overall detail, detail of benefits, and detail of risk. Forms with a high level of overall detail described how body systems were affected with specific, concrete examples of both risks, benefits, and needed preventative measures, while forms with a low level of overall detail consistently lacked specific examples of how treatments may affect the patient. Similarly, forms with a high level of detail of benefits specified clearly how reproductive and sexual organs will change in order to produce desired changes in gender presentation while forms with a low level of detail of benefits stated that treatments are gender-affirming and will cause changes to that effect, without specifying exactly what changes will occur. Lastly, forms with a high level of detail of risk specified changes that may result in adverse effects, while forms with a low level of detail of risk generally stated that body systems or biological processes may be affected without further discussion. Each type of form (PS, MH, FH) included a combination of forms with low detail and high detail, with more forms demonstrating a high level of detail overall (13 v 8). Forms that generally exhibited a low level of detail in discussion of risks also exhibited low level of details overall. All PS consent forms were less detailed than MH and FH forms both overall and in their description of risks and desired effects.

There was significant variation in the tone and presentation of information. Ten forms appeared to be constructed in a way that prioritized protection against legal culpability: “Please read the possible risks and effects listed below. It's important that you understand all this information…” (PS6) without emphasizing ongoing dialogue and conversation. Most forms (*n* = 7) oriented towards legal protection of clinicians also had lower levels of detail in all domains (risk, desired effects, and overall). Additionally, these forms used more complicated language and did not appear to be adapted for younger adolescents' expected health literacy or baseline sexual and reproductive health knowledge: “I understand that taking testosterone does not make me immune to gynecologic problems and I understand the recommendation to continue routine gyn care for the screening of cancer and sexually transmitted diseases” (MH5).

Eleven forms appeared to be more oriented towards education of patients/parents and open dialogue between clinicians and patients: “If any questions arise, either about this consent, or while taking hormone therapy, do not hesitate to ask your provider” (FH4). Forms that prioritized the relational aspects of communication and education of patients appeared to make fewer assumptions about prior medical knowledge. These forms explained concepts such as fertility and changes to sexual organs and behavior with more detail and less medical terminology. Three of the 11 forms that were determined to prioritize education and discussion were written in a manner that centered the adolescent patient and their goals. One form noted at the top of the form that the goals of the consent form were education, discussion, and assent from the pediatric patient. Few forms required the adolescent to sign in addition to the legal guardian to verify their understanding of the proposed treatment and its effects.

### 3.2. Changes in sexual and reproductive organs and functioning

**Figure 1:**
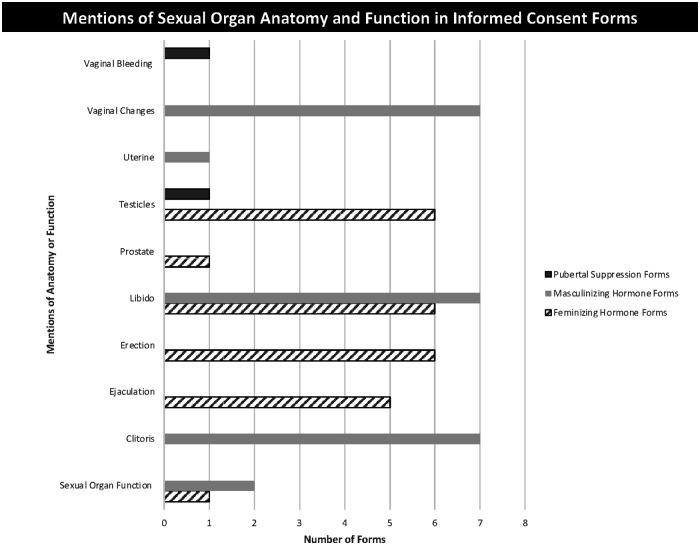
Mentions of sexual organ anatomy and function in informed consent forms.

Descriptions of expected changes in sexual organs were included in all consent forms. Most forms (*n* = 18) also included descriptions of whether changes are expected to be permanent or temporary. Some forms (*n* = 5) included a timeline for anticipated changes. Topics covered included breast growth or lack thereof, testicular changes, and changes in the uterus and vagina, however not all consent forms contained each of these topics (Figure 1). The specific topics varied appropriately according to the type of consent form, including information about the relevant sexual and reproductive organs based on sex-assigned-at-birth (i.e., descriptions of testicular changes were included only in feminizing consent forms). However, even within groups, there was considerable variability in the topics covered. Only a few pubertal suppression consent documents included specific information about the lack of breast development (“For natal females, pubertal changes that would not occur or progress while on treatment include development of breasts” (PS7)), while others referenced lack of pubertal progression more broadly (“Puberty Blockers are used to help temporarily suspend or block the physical changes of puberty” (PS6)).

The amount of information and level of detail provided about the types and extent of changes varied within and across consent form types. Most pubertal suppression consent forms referred generally to the fact that pubertal suppression medications will “temporarily suspend or block the physical changes of puberty for my child “ (PS1), while only a few provided more explicit details about specific changes in sexual and reproductive organs “and development of the ovaries and uterus which causes menstruation” (PS7). “These features include breasts, menses, testicular enlargement, and/or a deeper voice” (PS3).

All MH and FH consent forms included a description of expected breast changes, but this ranged from a simple statement such as, “Breast tissue development occurs and should be considered permanent once it develops.” (FH7) or “You may lose fat from breasts” (MH1) to more detailed information, “Breast size varies across all women. Some of this is genetic and somewhat predictable based on the size of the breasts of a mother, sisters, or aunts. Breasts may look smaller on a broad chest” (FH2).

Changes in sexual functioning were not included on any PS consent forms, but most FH and MH forms included information about changes to libido while on GAHs, such as “I know that I may want to masturbate less or have sex less and may find it harder to ejaculate when I do” (FH3) or, “More sex drive” (MH6). Most, but not all FH consent forms provided descriptions of expected changes in erections and ejaculation, “Decreased strength of erections or inability to get an erection” (FH2), including descriptions of changes in frequency and efficacy of erections as well as reduction in ejaculate. Vaginal changes were noted on all masculinizing consent forms, but only some forms (*n* = 4) linked these changes to sexual functioning: “Vaginal dryness and itching which can cause pain with vaginal penetration” (MH5). Clitoral enlargement was included as a potentially permanent change induced by testosterone in all MH consent forms with some including descriptions of the amount of growth expected, but no forms included changes in expected sexual function related to clitoral changes.

### 3.3. Pregnancy and fertility information

**Figure 2:**
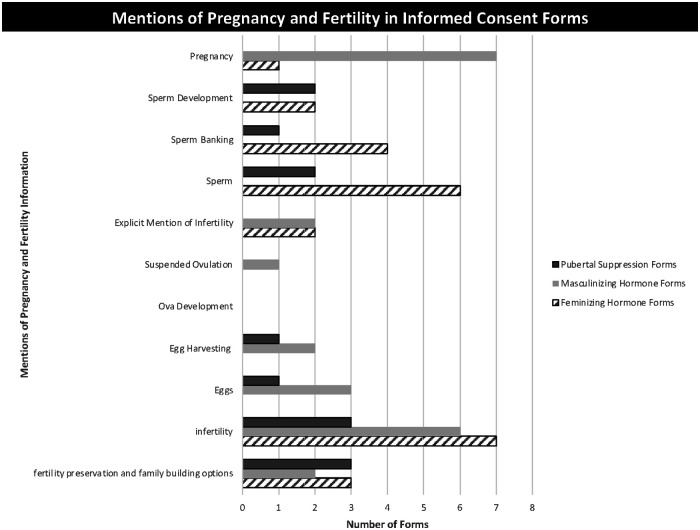
Mentions of pregnancy and fertility in informed consent forms.

Uncertainty regarding fertility outcomes for TNB youth and the potential for gender affirming treatments to affect future fertility occasioned a range of potentially incongruent information about pregnancy and fertility in consent documents; this included the need for contraception despite the anticipated impact of estrogen on sperm production, the need to consider fertility preservation options, and the potential for the fertility effects of pubertal suppression to become permanent if MHs or FHs are initiated without allowing puberty to progress (Figure 2).

All but one FH consent form included information about the need to protect against pregnancy in partners capable of bearing children, with some forms (*n* = 4) requesting initials next to a statement of understanding such as:

“I understand that estrogen may alter my fertility (ability to produce viable sperm), but that it is also not a contraceptive method. I understand that it is imperative that if I have vaginal sex with a biological woman (person with a uterus), I must use a barrier method to prevent an unintended pregnancy in my partner” (FH5).

Most forms (*n* = 14) included language about infertility and pregnancy prevention within the same paragraph or section:

“But I know that it's also possible that my sperm could still mature even while I am taking hormones. So, I know that I might get someone pregnant if we have vaginal intercourse and we don’t use birth control……I know this treatment may (but is not assured to) make me permanently unable to make a woman pregnant.“ (FH3).

MH consent forms included similar language about the need to prevent pregnancy while taking GAHs, with the inclusion of simple straightforward statements such as, “Testosterone is NOT birth control “ (MH4) and longer explanations, “Even with cessation of periods, pregnancy is possible while on testosterone. A barrier method of birth control is advised if engaging in sex where semen could enter the vagina or uterus” (MH7).

Fertility considerations and preservation options were discussed in all FH and MH forms and some PS consent forms. Language used to describe fertility preservation options was variable, with one form using language that was encouraging: “Sperm banking is a viable option for preserving reproductive options…Banking is encouraged for all males post puberty prior to the initiation of estrogen therapy” (FH7). Language describing FP in a different form was discouraging, “This means that my child would have to stop puberty blockers and complete their biological puberty… This process could take several years and there would be no guarantee of fertility.” (PS6). Lastly some forms (*n* = 3) used neutral language: “Some people choose to bank some of their sperm before starting hormone therapy.” (FH2).

### 3.4. Sexually transmitted infections

All MH consent forms contained general information about sexually transmitted infections, while few FH forms and no PS forms included such information. Most forms (*n* = 13) contextualized the increased or ongoing risk of sexually transmitted infections within a conversation about contraceptive use or biological changes that may contribute to an increased risk of STIs, such as cervical thinning and vaginal dryness. Forms that contextualized this risk generally ranked as having a high level of detail when describing risk. One example is seen in this masculinizing form (MH2): “I know taking testosterone can thin the tissue of my cervix and the walls of my vagina. This can lead to tears or abrasions during vaginal sex or play with a male or female partner. These tears increase my risk of getting a sexually transmitted infection, including HIV.”

Similar examples are found in feminizing forms (FH6):

I know that in addition to periodic checks from my provider, I must also treat my body with respect…This also means keeping my partners and myself safe, when and if I choose to have sex with others, by using condoms or methods to keep me safe from sexually transmitted infections (STIs).

Of the forms that discussed STIs, some included information about HIV risk in both sexual and non-sexual (needle-sharing) contexts. All masculinizing forms explicitly mentioned HIV, while few feminizing forms and no pubertal suppression forms explicitly mentioned HIV. Of these ten mentions of HIV, four of them related to contraction of HIV through needle-sharing as opposed to sexual contact.

### 3.5. Cancer risk

**Figure 3:**
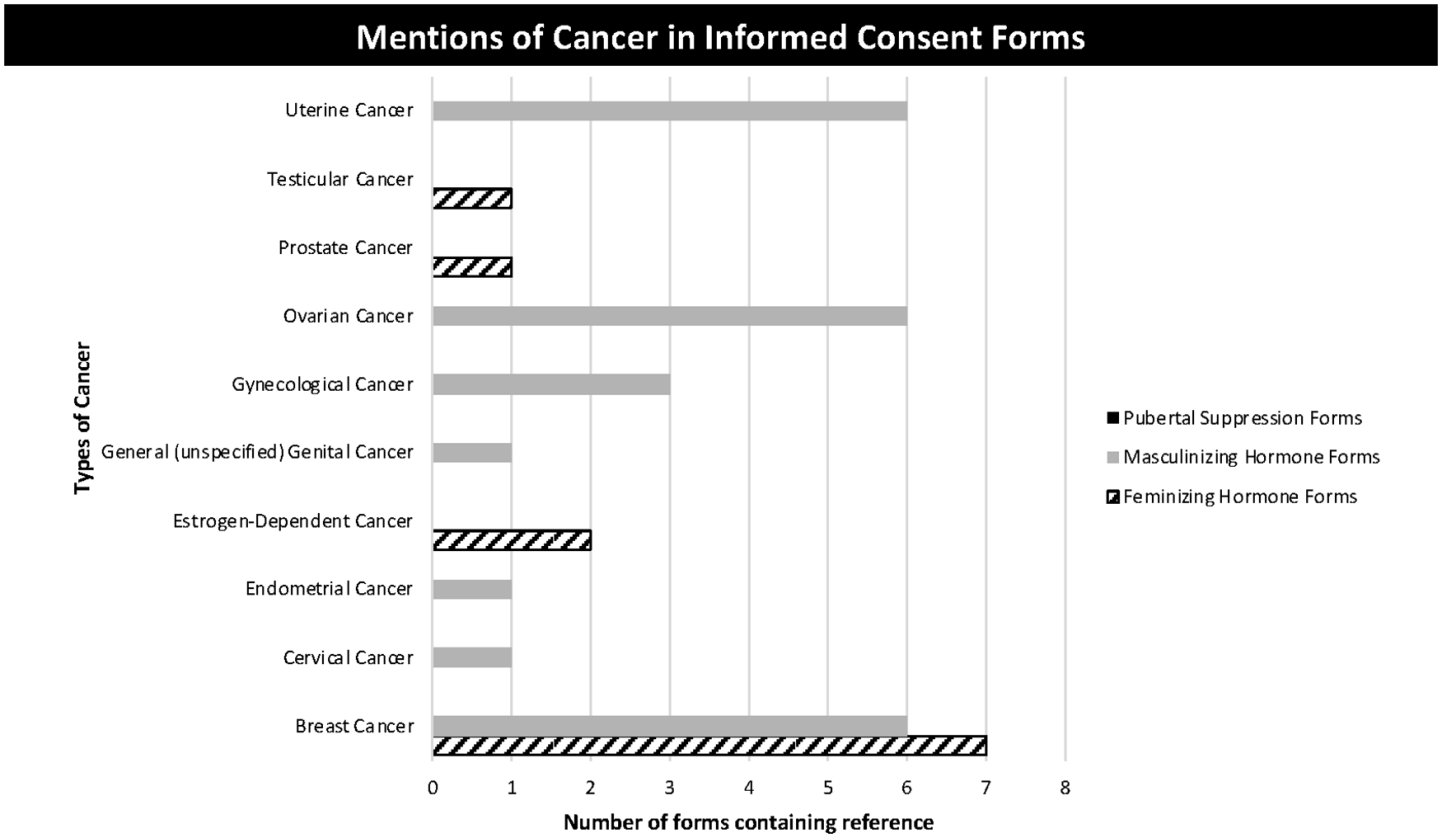
Mentions of cancer in informed consent forms.

A mention of cancer risk was included in nearly all MH and FH consent forms. Certain types of cancer were exclusive to MH consent forms or FH consent forms as appropriate for the reproductive organs expected for a particular sex-assigned-at birth (Figure 3).

Cancer risk was addressed only in FH and MH consent forms, however, not all forms addressed the relevant organs—for example, only 1 MH consent form addressed the risks of endometrial cancer and cervical cancer, while all MH consent forms included the risk of ovarian cancer. Similarly, only 1 FH consent form discussed testicular cancer and prostate cancer. Most MH and FH consent forms discussed the risk of breast cancer. Breast cancer was also often discussed in a separate section from the general discussion of cancer risk and was contextualized by discussion of changes in breasts and breast tissue. Discussion of cancer risk was highly entrenched in language of uncertainty, as exemplified by the following statement: “I know my body can turn testosterone into estrogen and that no one knows if that could increase the risk of cancers of the breast, the ovaries, or the uterus.” (MH7). This language was used consistently regardless of form category or cancer type.

## 4. Discussion

Our content analysis of 21 informed consent documents for youth seeking PS, MH, or FH found substantial variability in the inclusion and contextualization of SRH content highlighting potential limitations in using consent forms for providing SRH information. Despite the inclusion of similar broad content categories of SRH (changes in sexual organs, pregnancy and fertility information, cancer risk; and STIs) there was considerable variability in the level of detail, description of uncertainty, and tone of the information provided to TNB youth *via* informed consent documents. Consent forms for the use of pubertal suppression were the least detailed with respect to risks and benefits.

Our sample was limited to a convenience sample of consent forms available online and uploaded by English speaking providers within the United States who were eligible for a larger online survey project resulting in a small sample size and likely does not represent the larger international community of clinicians providing care to TNB youth. Clinics that use a verbal informed consent process but do not rely on a written consent form are not represented in this data set. Consent forms often undergo cyclic revisions, so it is possible that substantial additions and/or deletions were made after the forms were submitted to our online survey. Because the WPATH SOC 8th (22) edition was released after data analysis occurred, changes in SRH-related recommendations are not represented.

Informed consent in clinical practice operationalizes important ethical principles (e.g., respect for persons, beneficence, and nonmaleficence) and written forms are commonly used to document the disclosure of adequate information (including risks, benefits, and alternatives), patient/surrogate understanding, and a voluntary choice to proceed with the proposed treatment. Relevant laws and local (hospital, clinic) policies may also affect what information is included in consent forms and how they are utilized (23, 24). The role of informed consent forms in pediatrics have been more comprehensively studied in the research setting, documenting inadequate participant understanding and emphasizing efforts to improve comprehension beyond the use of written forms (25–28). Studies evaluating the efficacy of consent forms in clinical care also highlight the inadequacy of forms alone to ensure patients have adequate information, understanding, and autonomy in a shared-decision making process (29–32).

A number of articles have explored the theoretical components and ethical implications of informed consent, mental health letter requirements, and the decision making process for both TNB adults and youth (33–37). The “Informed Consent Model” which “allows for clients who are transgender to access hormone treatments and surgical interventions without undergoing mental health evaluation or referral from a mental health specialist” (38) has replaced a “diagnostic model” or “gatekeeping model” for adults seeking gender affirming care and does not require any standardized documentation or consent form (38, 39). For youth, guidelines recognize differences in legal age of consent and emphasize informed consent as a process and practice of shared decision making, “Ideally, treatment decisions should be made among the adolescent, the family, and the treatment team” (13), but do not provide guidance about standardizing or documenting youth participation or parental involvement. Studies examining the decision making process for TNB youth seeking gender affirming care note variability in power dynamics, parental support, and factors supporting informed decision making (40–44). Details about provider informed consent practices in various settings is still limited (43, 45), with only one study assessing TNB youth's decision-making ability that notes specifics about the informed consent process in two Dutch gender-identity clinics indicating that the process is standardized, occurs over several sessions (mean of 8.8 months) and concludes with parents and youth signing an informed consent document (43).

Clinical guidelines (12, 13) used by medical teams caring for TNB youth include a large amount of information pertaining to the specific sexual and reproductive health outcomes of youth receiving PS and gender affirming hormones. These guidance documents are lengthy and allow for ample discussion of the supporting data and inclusion of conflicting studies. They also rely heavily on medical jargon and assume readers have a sizeable scientific knowledge base. Distilling them into an informed consent document that is both informative and readable is challenging and not standardized across clinical sites, likely accounting for much of the variability across consent forms. Our study suggests that informed consent forms do not likely capture the full range of topics and conversations that occur between a provider, TNB youth, and their parents but the variability in content, the readability, tone of information presentation, and level of detail with respect to risks and benefits across analyzed forms suggests potential disparities in access to SRH information. Certain desired and adverse effects of pubertal blockers and GAH, such as changes in sexual function, gender presentation, menstrual patterns, fertility, and cancer risk should be enumerated in these informed consent documents more explicitly. This information should be presented in a way that is readable and digestible by youths seeking treatment and their parents. While informed consent forms can be an effective tool in engaging both youth and parents in decision making process, standardized forms do not recognize difference in informational needs that may exist between parents and child or between families from different backgrounds. Describing complex treatments in textual forms may not necessarily enable true informed consent, and additionally is not a reflection of conversations around informed consent that may be facilitated by the forms themselves; however, forms that are more accessible both in regard to readability and comprehensiveness empower TNB youths and their parents to engage more actively in their care.

Informed consent is an ongoing process (29), involving the patient, parent or legal guardian, and clinician that does not terminate with a signed document. Many SRH topics may require subsequent disclosure of information in addition to that provided in a consent form, or reinforcement of information that has already been provided. For example, providing HIV risk assessment, prevention, testing strategies and pre-exposure prophylaxis information likely require more in-depth information than can easily be provided in the context of starting GAHs. Additionally, while the effects of gender affirming care on fertility and FP methods were mentioned in all FH and MH consent forms, whether a form was perceived as encouraging, discouraging, or neutral may depend on the availability of local fertility resources (reproductive specialists) and insurance coverage (some states have considered laws that mandate coverage for fertility preservation (46)). PS forms in particular require significantly more detail with respect to desired and adverse effects and the prevention measures that should be taken in almost all realms of SRH, including but not limited to descriptions of concrete changes in sexual function, pubertal development, fertility, and cancer. The use of encouraging or discouraging language, as demonstrated on a number of informed consent forms in our study, should be avoided in value or preference sensitive treatment discussion and documentation.

Other research has explored the use of multimodal or decision support aids to improve informed consent in pediatric treatment. Multimedia aids have been found to improve participant knowledge more effectively than informed consent documents alone but are still no substitute for discussions about the risks and benefits of care that occur between pediatric patients, their guardians, and their care team (47). TNB youth need access to SRH that is affirming, inclusive, and accurate (48–50), and goes beyond the information needed, regardless of mode of presentation, to provide informed consent to PS, MH or FH.

Some consent forms sought to include more robust details about SRH topics but the benefit of presenting information in this way is unclear. Future research should further examine the role informed consent documents play in TNB youth's understanding of specific SRH information as well as whether it promotes a shared decision-making process for TNB youth and their parents. The role of decision aids and other ways of communicating complex SRH information to support decision making should also be further explored.

Informed consent documents for TNB youth seeking gender affirming medical interventions demonstrated consistency in the inclusion of certain key effects of PS, MHs, and FHs on sexual and reproductive organs, their functioning, risk for cancer and sexually transmitted infections. However, additional analyses and comparisons across and within categories revealed important differences in level of detail, uncertainty, and tone. The role of consent forms in fostering TNB youth's understanding of complex SHR information when initiating PS and GAHs needs further clarification and development.

## Data Availability

The raw data supporting the conclusions of this article will be made available by the authors, without undue reservation.
